# Supplementary material for: Cellular and Immunological Analysis of 2D2/Th Hybrid Mice Prone to Experimental Autoimmune Encephalomyelitis in Comparison with 2D2 and Th Lines
Source: Int J Mol Sci. 2024 Sep 13;25(18):9900. doi: 10.3390/ijms25189900 (PMC11432411; doi:10.3390/ijms25189900)
Supplement: Supplementary file 1 [file ijms-25-09900-s001.zip › ijms-3032397-supplementary.pdf]

International Journal of
Molecular Sciences

MDPI

Supplementary data; methods

Evaluation of different lymphocytes in samples of different mouse tissue

The relative content of different lymphocytes in various organs of mice was determined by flow cytometry as in [1]. The mice's peripheral blood was obtained after standard decapitation of mice, and sodium citrate was added to the blood to prevent coagulation. After leptocytes counting, 500 thousand cells in 150 μL were used for cytometric analysis. Cells were mixed with monoclonal Abs in the dark for 25 min, and then red cells in samples were lysed for 25 min using a 10-fold volume of RBC lysis buffer (Biolegend, San Diego, CA, USA). Then, cell specimens were centrifuged for 12 min and washed using 500 μL of PBS containing 1% sodium azide and 0.02% EDTA. After centrifugation, 55 μL of PBS was added to the cell sediment and analyzed on a flow cytometer.

Lymphocytes were obtained from five organs. The bone marrow was collected by washing the femoral cavity. Lymph nodes and thymus samples were meticulously homogenized using a glass homogenizer. Great particles were removed, and lymphocytes were resuspended using their passing through a needle of disposable syringe. Spleen cells were prepared by treating the organ using a medium-filled syringe through punctures of the spleen stroma. This approach allows obtaining splenocytes containing no impurities of the organ stroma. Cells were washed trice using centrifugation with 5 ml of RPMI-1640 medium for 12 min at 1500 pm. After additional centrifugation, RPMI-1640 medium (1 mL) containing 10 mM HEPES, 2 mM L-glutamine, 100 μg/mL benzylpenicillin, 10% fetal bovine serum, 80 μg/mL gentamicin, and 0.5 mM 2-mercaptoethanol, was added to the pellet, and the lymphocytes were counted. 500 thousand lymphocytes were used in 100 μL of PBS containing fetal bovine serum (10%) and the conjugates of various specific monoclonal Abs to analyze the cell content in different organs extracts. To analyze the relative number of various lymphocytes, specific antiCD45-BV510 (Biolegendcat # 103138), antiCD3-FITC (Biolegendcat # 100204), antiCD4-PerCP (Biolegendcat # 100432), antiCD8alpha-APC (Biolegendcat # 126614), and antiCD19-PE (Biolegendcat # 115508) antibodies (Biolegend, San Diego, California, USA) were used. All analysis were done according to the manufacturer's recommendations. Cells were incubated for 25 min with monoclonal antibodies and then washed by centrifugation after adding 500 μL of PBS containing EDTA and sodium azide. After centrifugation, 50 μL of PBS was added to the pellet, and the mixture obtained was used for analysis using the BD FacsVerse flow cytometer (BD Biosciences, SanJose, CA, USA). At least 1×105 events were collected for each preparation. Gating was calculated as follows: the total lymphocytes population was isolated according to granularity size of the cells, the leukocyte population was determined using the CD45+ pan-leukocyte marker, and populations of CD3+ and CD3– leukocytes were isolated. In the CD3+ leukocyte population (T cells), CD4+ and CD8+ T cells were determined, and in the CD3– leukocyte population, the content of CD19+ B cells was estimated.

1. Aulova, K.S.; Urusov, A.E.; Toporkova, L.B.; Sedykh, S.E.; Shevchenko, Y.A., Tereshchenko, V.P.; Sennikov, S.V.; Budde, T.; Meuth, S.G.; Orlovskaya, I.A.; Nevinsky, G.A. Catalytic antibodies in the bone marrow and other organs of Th mice during spontaneous development of experimental autoimmune encephalomyelitis associated with cell differentiation. Mol. Biol. Rep. 2021, 48, 1055-1068.
